# The impact of common polymorphisms in *CETP* and *ABCA1* genes with the risk of coronary artery disease in Saudi Arabians

**DOI:** 10.1186/s40246-016-0065-3

**Published:** 2016-03-02

**Authors:** Cyril Cyrus, Chittibabu Vatte, Awatif Al-Nafie, Shahanas Chathoth, Rudaynah Al-Ali, Abdullah Al-Shehri, Mohammed Shakil Akhtar, Mohammed Almansori, Fahad Al-Muhanna, Brendan Keating, Amein Al-Ali

**Affiliations:** Institute for Research and Medical Consultation, University of Dammam, P.O.Box 1982, Dammam, 31441 Kingdom of Saudi Arabia; King Fahd Hospital of the University, University of Dammam, P.O.Box 4001, Al-Khobar, 31952 Kingdom of Saudi Arabia; Department of Pediatrics, Perelman School of Medicine, University of Pennsylvania, Philadelphia, PA USA

**Keywords:** Gene polymorphism, CAD, *CETP*, *ABCA1*, TaqMan Assay

## Abstract

**Background:**

Coronary artery disease (CAD) is a leading cause of morbidity and mortality worldwide. Many genetic and environmental risk factors including atherogenic dyslipidemia contribute towards the development of CAD. Functionally relevant mutations in the dyslipidemia-related genes and enzymes involved in the reverse cholesterol transport system are associated with CAD and contribute to increased susceptibility of myocardial infarction (MI).

**Method:**

Blood samples from 990 angiographically confirmed Saudi CAD patients with at least one event of myocardial infarction were collected between 2012 and 2014. A total of 618 Saudi controls with no history or family history of CAD participated in the study. Four polymorphisms, rs2230806, rs2066715 (*ABCA1*), rs5882, and rs708272 (*CETP*), were genotyped using TaqMan Assay.

**Results:**

*CETP* rs5882 (OR = 1.45, *P* < 0.005) and *ABCA1* rs2230806 (OR = 1.42, *P* = 0.017) polymorphisms were associated with increased risk of CAD. However, rs708272 polymorphism showed protective effect (B1 vs. B2: OR = 0.80, *P* = 0.003 and B2B2 vs. B1B1: OR = 0.68, *P* = 0.012) while the *ABCA1* variant rs2066715 was not associated.

**Conclusion:**

This study is the first to report the association of these polymorphisms with CAD in the population of the Eastern Province of Saudi Arabia. The rs5882 polymorphism (*CETP*) showed a significant association and therefore could be a promising marker for CAD risk estimation while the rs708272 polymorphism had a protective effect from CAD.

## Background

Coronary artery disease (CAD) is one of the leading causes of morbidity and disability and the most common cause of mortality worldwide equally among men and women. CAD is a disease burden in both high- and low-income countries [1, 2]. A study conducted on 17,232 people from Saudi Arabia revealed that 5.5 % had been diagnosed with CAD, with a higher prevalence in urban populations (6.2 %) compared to rural populations (4 %) [3]. Platelet aggregation and thrombus formation following the rupture of coronary atherosclerotic plaque is the major cause of myocardial infarction (MI) [4–6].

Many extrinsic and intrinsic risk factors, including hypertension, dyslipidemia, obesity, smoking, age, lack of exercise, and diabetes, are established risk factors for MI [7]. Atherogenic dyslipidemia is characterized by abnormal levels of triglycerides, low- and high-density lipoprotein (LDL-C and HDL-C) [8–10]. Functionally relevant mutations in the dyslipidemia-related genes and gene encoding enzymes involved in the reverse cholesterol transport system have been reported to be associated with high-density lipoprotein-cholesterol (HDL-C) levels [11–13]. Epidemiological and clinical studies have demonstrated a contradictory association between HDL-C concentrations and cardiovascular risk [10, 14, 15]. The anti-atherogenic effect of HDL-C may act through several mechanisms, such as anti-oxidation of low-density lipoprotein-cholesterol (LDL-C) and anti-inflammation and inhibition of vascular endothelial cell apoptosis.

The reverse cholesterol transport system plays a vital role in these processes [16], as it is involved in the transportation of cholesterol from the peripheral tissues to the liver, where cholesterol is secreted into bile. ATP-binding-cassette A1 (ABCA1), apolipoprotein A-1 (ApoA-1), and cholesteryl ester transfer protein (CETP) play important roles in the reverse cholesterol transport system [17]. Certain *ABCA1* polymorphisms have been reported to be associated with HDL-C concentrations, which in turn indicate increased cardiovascular risk [18]. The potential atherogenicity of CETP relates to its ability to transfer cholesteryl esters from the anti-atherogenic HDLs to the pro-atherogenic VLDL and LDL proteins. Mutations in the *CETP* gene give rise to less functional protein, which reduces the transfer of cholesteryl esters, and consequently HDL levels are elevated [19]. *ABCA1* and *CETP*, variants including rs2230806, rs2066715, and rs5882, have been associated with increased HDL-C concentrations and rs708272 with a decreased risk for CAD [20, 21].

The objective of the present study is to evaluate the association of the two *ABCA1* polymorphisms, rs2230806 [R219K: c.656G>A (p.Arg219Lys)] and rs2066715 [V825I: c.2473G>A (p.Val825Ile)], and two *CETP* polymorphisms, rs5882 [V422I: c.1264G>A (p.Val422Ile)] and rs708272 [TaqIB: c.118+279G>A], with the risk of CAD in the population of the Eastern Province of Saudi Arabia.

## Results

Demographical and clinical data of cases and the control group, including age, sex, clinical manifestations, and biochemical parameters, are shown in Table 1. Patients were classified into subgroups based on their hypertension and diabetes status. Hypertension and diabetes were more prevalent in the patient group compared to the control group.

**Table 1:** Demographic and clinical characteristics of study subjects

Baseline characteristics	Cases (*N* = 990)	Controls (*N* = 618)	*P* value
Age (years)	58.37 ± 12.91	54.8 ± 8.5	*<0.0001* ^a^
Sex M:F	708:282	423:195	0.19^b^
Total cholesterol (mg/dl)	170.84 ± 48.39	171.58 ± 43.25	0.50^a^
LDL cholesterol (mg/dl)	103.82 ± 40.58	107.25 ± 35.35	*0.005* ^a^
HDL cholesterol (mg/dl)	36.19 ± 9.88	50.22 ± 23.89	*<0.0001* ^a^
Clinical Characteristics			
Hypertension, *n* (%)	688 (69.5)	74 (12.0)	*<0.05* ^b^
Diabetes, *n* (%)	577 (58.3)	87(14.1)	*<0.05* ^b^

^a^Student’s *t* test

^b^Chi-square test

All genotype frequencies of the control group were consistent with Hardy-Weinberg equilibrium. The distribution of analyzed genotype polymorphisms are shown in Table 2. Since all the four SNPs had a G>A transition substitution, the genotypes are denoted with the amino acid change, except for Taq1B alleles, which are designated by B1 and B2. The genotype analysis showed overall heterozygous polymorphism predominance in rs2230806 of *ABCA1*, rs5882, and rs708272 of *CETP* (Table 2).

**Table 2:** Association between *ABCA1* and *CETP* genotypes and alleles with CAD

SNP	Genotype/alleles	Cases (*N* = 990)	Control (*N* = 618)	OR (95 % CI)	*P* value
rs2230806 (R219K)	RR	291	195	Ref	
	RK	473	317	0.9 (0.79–1.25)	0.999
	KK	226	106	1.42 (1.06–1.91)	*0.017*
	RK+KK	699	423	1.10 (0.89–1.37)	0.359
	R(G)	1055	707	Ref	
	K(A)	925	529	1.17 (1.01–1.35)	*0.029*
rs2066715 (V825I)	VV	945	597	Ref	
	VI	45	21	1.35 (0.79–2.29)	0.26
	II	0	0	–	–
	VI+II	45	21	1.35 (0.79–2.29)	0.26
	V(G)	1935	1215	Ref	
	I(A)	45	21	1.34 (0.79–2.26)	0.27
rs5882 (V422I)	VV	178	147	Ref	
	VI	523	297	1.45 (1.12–1.88)	*0.005*
	II	289	174	1.37 (1.02–1.82)	*0.031*
	VI+II	812	471	1.42 (1.11–1.82)	*0.005*
	V(G)	879	591	Ref	
	I(A)	1101	645	1.14 (0.99–1.32)	0.058
rs708272 (TaqIB)	B1B1	376	183	Ref	
	B1B2	454	321	0.68 (0.54–0.86)	*0.001*
	B2B2	160	114	0.68 (0.50–0.92)	*0.012*
	B1B2+B2B2	614	435	0.68 (0.55–0.85)	*0.0006*
	B1(G)	1206	687	Ref	
	B2(A)	774	549	0.80 (0.69–0.92)	*0.003*

The *CETP* rs708272 polymorphism showed a significantly lower risk for CAD (B1B2+B2B2 vs. B1B1: OR = 0.68, 95 % CI 0.55–0.85, *P* = 0.0006 and B2B2 vs. B1B1: OR = 0.68, 95 % CI 0.50–0.92, *P* = 0.012). There was also a significant variation of B1B2 genotypes among patients and controls (OR = 0.68, 95 % CI 0.54–0.86, *P* = 0.001). Genotyping for the rs5882 polymorphism in *CETP* exon-14 showed that the frequency of VI genotype was higher in cases than in controls (52.8 vs. 48.0 %). Our analysis revealed that *CETP* rs5882 polymorphism is associated with an increased risk of CAD in our Saudi population study dataset (VI+II vs. VV: OR = 1.42, 95 % CI 1.11–1.82, *P* = 0.005; II vs. VV: OR = 1.37, 95 % CI 1.02–1.82, *P* = 0.031). Allele frequency analysis of the B2 allele of rs708272 of *CETP* (OR = 0.80, 95 % CI 0.69–0.92, *P* = 0.003) and the K allele of rs2230806 of *ABCA1* (OR = 1.17, 95 % CI 1.01–1.35, *P* = 0.029) showed a significant difference between the two tested groups (Table 2). The mutant KK genotype of rs2230806 of *ABCA1* is found to be associated with an increased risk of CAD (RR vs. KK: OR = 1.42, 95 % CI 1.06–1.91, *P* = 0.017). There were no significant differences in allele and genotype frequencies of rs2066715 polymorphisms in *ABCA1* between the patient and control groups. The power of the study observed was 100 % for protective effect at odds ratio of 0.5 and 94.9 % at 0.7 for TaqIB, and for the other three SNPs (R219K, V825I, and I405V), the results ranged from 45.48 to 96.9 % for an odds ratio of 1.2–1.5.

A joint analysis of two SNPs of both *ABCA1* and *CETP* is shown in Table 3. All the combinations of the *CETP* variants exhibited no association, except B1B1+VI (OR = 1.7, 95 % CI 1.0–2.9, *P* = 0.048). On the other hand, for *ABCA1*, RK+VV and RK+VI lacked an association (Table 3). A sex-based analysis revealed a higher frequency of B1B1 genotype in men and women with CAD compared to their respective controls (Table 4). There was no statistical significance in the distribution of *ABCA1* genotypes in the female cohort whereas in the male cohort genotypes KK (OR = 1.8, *P* = 0.001) of rs2230806 and VI (OR = 2.17, *P* = 0.041) of rs2066715 showed a significantly higher risk for CAD. In the *CETP*, rs5882 polymorphisms II (OR = 1.98, 95 % CI 1.38–2.85, *P* = 0.0002) revealed a significantly higher risk for CAD in the male cohort. In the male and female cohorts, rs708272 B1B2 and B2B2 genotypes showed a protective effect for CAD, respectively. The rs2230806 and rs2066715 of *ABCA1* did not show any significant association with an increased risk for CAD in the female cohort compared to the control subjects.

**Table 3:** Association between joint analysis of two SNPs of *ABCA1* gene and *CETP* gene with CAD

Gene	Genotype	Cases (*N* = 990)	Controls (*N* = 618 )	OR	95 % CI	*P* value
*ABCA1*	RR+VV	256	186	Ref		
	RR+VI	35	9	2.82	1.32–6.02	*0.007*
	RK+VV	463	305	1.10	0.86–1.39	0.419
	RK+VI	10	12	0.60	0.25–1.43	0.252
	KK+VV	226	106	1.54	1.14–2.08	*0.004*
*CETP*	B1B1+VV	46	31	Ref		
	B1B1+VI	180	71	1.70	1.0–2.90	*0.048*
	B1B1+II	150	81	1.24	0.73–2.11	0.41
	B1B2+VV	77	71	0.73	0.41–1.27	0.27
	B1B2+VI	269	175	1.03	0.63––1.69	0.888
	B1B2+II	108	75	0.97	0.56–1.66	0.913
	B2B2+VV	55	45	0.82	0.45–1.50	0.527
	B2B2+VI	74	51	0.97	0.54–1.74	0.939
	B2B2+II	31	18	1.16	0.55–2.42	0.692

**Table 4:** Association between *ABCA1* and *CETP* genotypes and CAD in male and female cohorts

Genotype		Male cohort				Female cohort			
		Cases (*n* = 708)	Control (*n* = 423)	OR (95 % CI)	*P* value	Cases (*n* = 282)	Control (*n* = 195)	OR (95 % CI)	*P* value
R219K	RR	212	137	Ref		79	58	Ref	
	RK	337	229	0.95 (0.72–1.24)	0.718	136	88	1.13 (0.73–1.74)	0.566
	KK	159	57	1.80 (1.24–2.61)	*0.001*	67	49	1.0 (0.60–1.65)	0.987
V825I	VV	676	414	Ref		269	183	Ref	
	VI	32	9	2.17 (1.02–4.60)	*0.041*	13	12	0.73 (0.32–1.65)	0.458
V422I	VV	123	103	Ref		55	44	Ref	
	VI	376	232	1.35 (0.99–1.84)	0.052	147	65	1.80 (1.10–2.96)	*0.018*
	II	209	88	1.98 (1.38–2.85)	*0.0002*	80	86	0.74 (0.45–1.22)	0.246
TaqIB	B1B1	261	124	Ref		115	59	Ref	
	B1B2	340	247	0.65 (0.49–0.85)	*0.002*	114	74	0.79 (0.51–1.21)	0.282
	B2B2	107	52	0.97 (0.65–1.45)	0.91	53	62	0.43 (0.27–0.71)	*0.0008*

## Discussion

CAD is a multifactorial disease mediated through a complex association of environmental and genetic factors with ethnicity demonstrated to be an important determinant of disease variability. The strength of the current study is the selection of CAD patients from similar ethnic backgrounds. In the present study, two common genetic variations in each *ABCA1* and *CETP* gene were studied with reference to their effect on CAD. We tested four SNPs, namely rs2230806 (R219K), rs2066715 (V825I), rs5882 (V422I), and rs708272 (*TaqIB*) for their association with CAD. *CETP*, located on chromosome 16q21, plays a crucial role in lipid metabolism, and numerous SNPs in this gene have been reported to alter the plasma HDL-C levels and function of CETP [22, 23]. Among the *CETP* SNPs, rs708272 is the one that is most studied. Therefore, we investigated the association of two SNPs of this gene and their risk for CAD in the Saudi population.

Our overall results showed that the heterozygous and mutant of rs708272 polymorphism may confer protection against CAD (B1B2: OR = 0.68, *P* = 0.001; B2B2: OR = 0.68, *P* = 0.012) while those of rs5882 increased the risk of CAD (VI: OR = 1.45, *P* = 0.005; II: OR = 1.37, *P* = 0.031). Earlier studies had suggested that the *CETP* variant rs5882 causes low CETP and is associated with higher HDL and possibly with increased CAD among hyper-triglyceridemia men [24, 25]. In contrast, in many recent studies, the rs5882 polymorphism lacked any association with CAD [26–29]. *ABCA1* encodes an important protein that facilitates the formation of HDL-C and regulates the efflux of lipids from peripheral cells into lipid-poor ApoA1 particles, stimulating reverse cholesterol transport. [30] The association between the *ABCA1* gene polymorphisms and CAD has been the focus for many studies [31–33]. The rs2230806 is the most common polymorphism of *ABCA1*; the possible role of rs2230806 in cardiovascular diseases is still debatable as numerous studies have reported divergent results [34, 35]. The results of our study revealed that the K variant of rs2230806 (*P* = 0.029) is associated with CAD, which is in line with Zargar et al. [36]. The rs2066715 of *ABCA1* is not associated with an increased risk of CAD. The frequency of the rare allele of rs2066715 (0.02) showed a unique distribution compared to the 1000 genome database (0.113) and Han Chinese (0.44) population [37]. The K allele frequency of rs2230806 (R219K) polymorphism in our study was 0.47 compared to 0.28–0.73 in earlier studies [18, 38, 39]. The K allele frequency reported in our study is in line with other reports, namely a European ancestry study which reported a K allele frequency of 0.26–0.46 and a study on Dutch men with proven CAD, which reported a frequency of 0.46 [40, 41].

An analysis of the effect of a combination of *ABCA1* genotypes on CAD showed that the RR+VI genotype was significantly associated with a high risk of CAD (OR = 2.82, 95 % CI 1.32–6.02, *P* = 0.007). However, an analysis of the effect of the combination of CETP genotypes on CAD showed no significant pattern. All other combinations lacked a significant association with an OR ranging from 0.73 to 1.24, except for B1B1+VI (OR = 1.7, *P* = 0.048). The prevalence of CAD was higher in males (M:F 708:282) than in females in the present study. Homozygous mutant and heterozygous of rs5882 in men and women were strongly associated with an increased risk of CAD, while the other two *ABCA1* polymorphisms showed no significant association with CAD (OR = 0.73–1.13) in the female cohort. Also, heterozygousity of rs708272 alleles was strongly associated with an increased risk of CAD (OR = 0.65, *P* = 0.002), a homozygous carriage of rs5882, the rarer variants II causing amino acid substitutions showed a strong association with an increased risk of CAD (OR = 1.98, *P* = 0.0002) among the male cohort. The *CETP* rs5882 polymorphism was found to be associated with an increased risk for CAD in the overall study population and also in the male and female cohorts, which is in contrast to a recent study which reported that this polymorphism is associated with a decreased risk of CAD and MI [42].

Studies conducted in other populations have correlated the genotypes of *ABCA1* and *CETP* and risk of CHD with respect to its effect on HDL-C. A study of 119 patients in Korea showed that the B1B1 genotype of the *CETP* Taq1B polymorphism was associated with low HDL-C levels in females and non-smoking males and may be an independent genetic risk factor for CAD [43]. In the present study, the B2 allele of the *CETP* rs708272 polymorphism was associated with a reduced risk of CAD mediated by elevated HDL-C concentrations. However, Borggreve et al. in their prospective population-based study (PREVENT study) on 8141 Caucasians demonstrated that the B2 and I alleles of the rs708272 (*TaqIB*) and rs5882 (V422I) of the *CETP* gene were not associated with a decreased risk for CAD, despite their HDL-C-raising effect suggesting that the risk may be independent of the gene’s influence on HDL-C levels [44]. Thus, the association of polymorphic *CETP* genotypes with a decreased cardiovascular risk seems to be independent of their effect on HDL-C levels [45–47].

## Conclusion

This study is the first to report the association of these polymorphisms with the development of CAD in a Saudi population. A significant association of *CETP* rs5882 and *ABCA1* rs2230806 polymorphism with CAD was observed marking these polymorphisms as risk factors. The rs708272 showed a protective effect for CAD, and rs2066715 of *ABCA1* gene lacked any association with CAD, whereas the joint effect of the *ABCA1* gene (RR+VI and KK+VV) conferred a higher risk for CAD. A sex difference subsists with a higher prevalence of CAD among males (M:F 708:282). Female heterozygous and male homozygous for the rs5882 were shown to have an increased risk of CAD.

## Methods

### Study population

Patients reporting to the cardiac clinic at King Fahd Hospital of the University, Al-Khobar, and other major hospitals in the Eastern Province of Saudi Arabia were screened, and angiographically confirmed CAD cases with at least one event of MI (*N* = 990) were enrolled in this study. A total of 618 age-matched normal Saudi controls with no history or family history of CAD were recruited from the blood banks of the same hospitals. This study was approved by the Ethical Committee of the University of Dammam. Signed written informed consent was obtained from all participants.

### Genotyping

Blood samples (5 ml) were obtained from 1608 subjects in EDTA-coated tubes and DNA was extracted using QIAamp DNA isolation kit (Qiagen, Germany) as per the manufacturer’s instructions. Allele-Specific TaqMan® PCR procedures were used to detect the genetic variants rs2230806, rs2066715, rs708272, and rs5882.

### Statistical analysis

The difference between cases and controls was evaluated using *t* test for continuous variables and Chi-square test for discrete variables. Allele frequencies were estimated by direct counting of the test allele divided by the total number of alleles. To assess the risk for CAD, odds ratio was determined by univariate analysis. All statistical analyses were performed using SPSS software (version19). The power of the study was calculated using online software sampsize.sourceforge.net.
